# Formation of a Unique Population of CD8+ T Lymphocytes after Adoptive Transfer of Syngeneic Splenocytes to Mice with Lymphopenia

**DOI:** 10.1134/S1607672921020137

**Published:** 2021-03-05

**Authors:** Yu. Yu. Silaeva, A. A. Kalinina, L. M. Khromykh, A. V. Deykin, D. B. Kazansky

**Affiliations:** 1 Core Facility Center, Institute of Gene Biology, Russian Academy of Sciences, Moscow, Russia; 2 Blokhin National Medical Research Center of Oncology, Ministry of Health of the Russian Federation, Moscow, Russia; 3 Center for Precision Genome Editing and Genetic Technologies for Biomedicine, Institute of Gene Biology, Russian Academy of Sciences, Moscow, Russia

**Keywords:** surrogate memory T cell, lymphopenia, TCR, CD44, CD62L, CD5, CD122, CD49d, CXCR3

## Abstract

Under conditions of lymphopenia, T lymphocytes proliferate and acquire a surface activation phenotype, which in many respects is similar to the phenotype of true memory T cells. We investigated the phenotypic features of the CD8+ T-cell population formed from donor lymphocytes after adoptive transfer of syngeneic splenocytes to sublethally irradiated mice. This population expresses markers CD44, CD122, CD5, CD49d and the chemokine receptor CXCR3. Thus, for the first time, the phenomenon of the formation of a population of T cells with signs of suppressive CD8+ T lymphocytes and true memory cells was demonstrated.

Under conditions of lymphopenia, populations of surrogate CD8+ memory T cells, which are phenotypically similar to the true memory cells (“memory-like” CD8+, T_ML_ cells), appear in the body [1–4]. Results of first experiments suggested that the T_ML_ population forming under conditions of lymphopenia repeats the phenotypic features of the true memory cells and can replace them in the immune response [5, 6]. However, evidence is accumulating that T_ML_ cells are similar to the true memory cells in terms of the expression profile of surface markers; however, there are principal differences between them. For example, the expression of chemokine receptors in T_ML_ cells differs from that in the true memory cells [7]. T_ML_ populations with immunosuppressive activity have been described [8]. Moreover, under conditions of lymphopenia, T-cell clones with receptors for high-affinity interaction with their own MHC molecules (essentially, the autoreactive T cells) proliferate and express surface markers of memory cells [9, 10]. In this work, we studied the phenotype of the population of CD8+ lymphocytes that is formed as a result of adoptive transfer of syngeneic splenocytes to recipient mice with lymphopenia after their sublethal irradiation.

Mouse lines C57BL/6 (K^b^I-A^b^D^b^) and C57BL/6-TgN(ACTbEGFP)1Osb (K^b^I-A^b^D^b^) (hereinafter, B6.GFP, https://www.jax.org/strain/003291), which were bred at the vivarium of Blokhin National Medical Research Center of Oncology, were used. In B6.GFP mice, GFP is expressed constitutively under the control of chicken beta-actin promoter and cytomegalovirus enhancer. No differences in the functioning of the immune system of the transgenic animals in comparison with the wild-type mice were detected, which allowed us to use this line in experiments. Female C57BL/6 mice were sublethally irradiated (4.5 Gy once) using an Agat-R instrument (Russia) (a source of γ-radiation Co^60^, an initial power 1.9 × 10^14^ Bq). The animals were withdrawn from the experiment on day 10 after irradiation, their spleens were extracted and homogenized in phosphate-buffered saline at 4°C. Splenocytes were precipitated by centrifugation (200*g*, 5 min). Erythrocytes were treated with lysis buffer (BD Pharmingen, United States). Mononuclear cells were washed three times with phosphate-buffered saline and used for staining with monoclonal antibodies and adoptive transfer.

We used monoclonal antibodies conjugated with the corresponding fluorescent labels: PerCP-Cy5.5—anti-CD8α (clone 53-6.7, BD Bioscience, United States), APC-Cy7—anti-CD62L (clone MEL-14, eBioscience, United States), APC—anti-CD44 (clone IM7, eBioscience), PE-Cy7—anti-CD3 (clone 145-2C11, eBioscience), PE—anti-CD122 (clone TM-β1, BD Bioscience), BV421—anti-CD5 (clone 53-7.3, BD Biosciences), BV421—anti-CXCR3 (clone CXCR3-173, BD Biosciences), and PE—anti-CD49d (R1-2, BD Biosciences).

Adoptive transfer was carried out as follows: nonimmunized C57BL/6 mice were irradiated at a dose of 4.5 Gy. Then, 24 h after irradiation, mice were intravenously (i.v.) injected with 1.5 × 10^7^ splenocytes from nonimmunized syngeneic animals or with phosphate-buffered saline (control). After 10 days, the splenocytes of the recipient mice were used for cytometric analysis. Splenocytes (3 × 10^6^ cells) were incubated with blocking antibodies Fc block (clone 2.4G2, BD Pharmingen, United States; 10 min, 4°C), stained with fluorescently labeled antibodies (40 min, 4°C), and analyzed with a FACS Canto II flow cytometer (BD Bioscience) using the FACSDiva 6.0 software (BD Bioscience). To characterize the populations of peripheral T cells, at least 10^6^ events were analyzed in each sample. Data were processed using the Flow Jo 7.6 software (TreeStar Inc., United States). The results were represented as the mean value ± standard error of the mean (*M* ± *SEM*). Statistical analysis was performed using the unpaired Student’s *t* test. Differences were considered significant at *p* ≤ 0.05.

A significant increase in the relative number of CD3+ lymphocytes in the spleen of the recipient mice was detected (Fig. 1a). In addition, the proportion of CD8+ T cells also significantly increased in the recipient mice as compared to the nonimmunized control mice and the sublethally irradiated animals (Fig. 1a). This means that the adoptive transfer of syngeneic splenocytes leads to the proliferation of primarily CD8+ T cells. This fact is consistent with the results of studies showing that the CD8+ cells require fewer stimuli for homeostatic proliferation as compared to the CD4+ T lymphocytes [11]. Almost all CD8+ T lymphocytes of both donor and recipient acquired the CD44+ phenotype, as expected under conditions of lymphopenia (Fig. 1b). It should be noted that the proportion of the potentially autoreactive CD8+CD44+CD5+ cells in the donor lymphocyte population was significantly higher than that among the recipient lymphocytes (Fig. 1b). The proportion of the CD8+CD122+ T cells among the donor T lymphocytes also considerably increased as compared to the analogous populations of the recipient, nonimmunized and sublethally irradiated mice (Fig. 1b). The proportion of T cells expressing CD49d in the donor cell population significant increased as compared to the recipient T cells (Fig. 1c). In addition, the relative number of donor T lymphocytes expressing the chemokine receptor CXCR3 was significantly higher than the proportion of such lymphocytes in the recipient T-cell population (Fig. 1c).

**Fig. 1.**
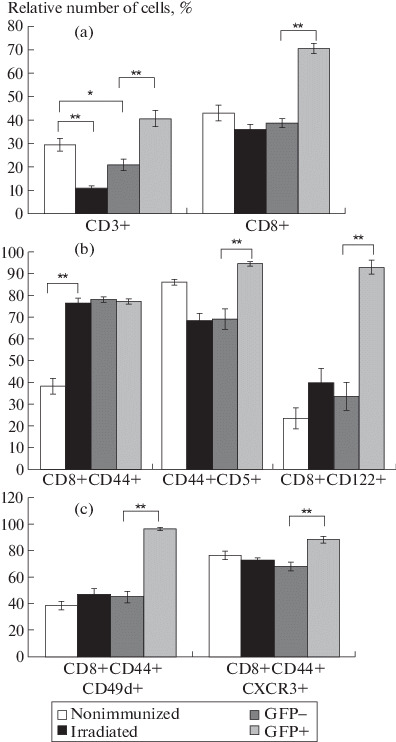
Relative number of T-lymphocyte populations in the spleen of sublethally irradiated recipient mice after adoptive transfer of syngeneic splenocytes. (a) Relative number of CD3+ and CD3+CD8+ T cells in the spleen of sublethally irradiated recipients. Data are presented for the control groups (nonimmunized, irradiated) separately for recipient (GFP–) and donor (GFP+) cells. Here and in Figs. 1a–1c, data were obtained in three independent experiments (four to six animals in each group). Statistical analysis was performed using Student’s *t* test (*p ≤ 0.05, **p  ≤  0.01). (b) Relative number of CD8+CD44+, CD44+CD5+, and CD8+CD122+ T cells in the spleen of sublethally irradiated recipients. (c) Relative number of CD8+CD44+CD49d+ and CD8+CD44+CXCR3+ T cells in the spleen of sublethally irradiated recipients.

Thus, the adoptive transfer of syngeneic splenocytes leads to the formation of a population of donor T cells with unique phenotypic characteristics—a simultaneous expression of markers of true memory cells and CD8+ suppressors—in the spleen of a sublethally irradiated recipient. We believe that the surface phenotype acquired by the donor T lymphocytes under conditions of lymphopenia may be associated with a deficiency or excess of signals received by T lymphocytes during the interaction of the T-cell receptor with intrinsic MHC–peptide complexes. We have shown that the competition for interaction with the intrinsic MHC–peptide complexes among T lymphocytes carrying the transgenic TCR β chain in mice of the 1D1b line leads to a change in the surface activation phenotype as compared to the T cells of the same animals expressing the endogenous β-chain of TCR [12]. In our experimental system, naive donor cells can benefit from a high level of expression of the CD5 molecule, especially given the fact that a considerable part of the recipient naive cells died as a result of sublethal irradiation. In addition, changes in the microenvironment of the donor T lymphocytes resulting from the adoptive transfer of splenocytes to the recipient venous bed may be of great importance in the formation of the discovered population. However, regardless of the causes of the appearance of such expression profile of surface markers, the most important question is the functional characteristics of the discovered population: are they capable of implementing a full-fledged immune response or are they a population of suppressor T cells? In the last variant, the obtained results may have a practical value for the practice of blood transfusion and bone marrow transplantation, because such populations may form in patients [13]. Therefore, the study of the functional characteristics of the discovered population is extremely important and will become the subject of our further research.
